# A social profile of deaths related to sickle cell disease in India: a case for an ethical policy response

**DOI:** 10.3389/fpubh.2023.1265313

**Published:** 2023-12-21

**Authors:** Sangeeta Chattoo, Dipty Jain, Nidhi Nashine, Rajan Singh

**Affiliations:** ^1^Department of Sociology, University of York, York, United Kingdom; ^2^Department of Pediatrics, Arihant Hospital, Nagpur, India; ^3^Council of Scientific and Industrial Research (CSIR), New Delhi, India; ^4^Indira Gandhi Government Medical College and Hospital, Nagpur, Maharashtra, India; ^5^Institute of Socio-Economic Research on Development and Democracy (ISERDD), New Delhi, India

**Keywords:** sickle cell, India, mortality, structural inequality, ethical policy

## Abstract

India accounts for 14.5 percent of the global SCD newborns, roughly over 42,000 a year, second to sub-Saharan Africa. Despite the availability of cheap diagnostic and treatment options, SCD remains a largely neglected disease within healthcare policy and practice. Epidemiological modeling based on small, often dated, regional studies (largely from sub-Saharan Africa) estimate that between 50 and 90 percent of affected children will/die before the age of 5 years. This premise, coupled with targets of reducing under 5 mortality (SDG 4), privileges public health interventions for screening and prevention of new births, undermining investments in long-term health and social care. This paper presents a retrospective, descriptive analysis of the socio-demographic profile of 447 patients diagnosed with sickle cell or sickle-beta thalassemia, who died following admission at a tertiary care entre in India. We used anonymized hospital records of 3,778 sickle cell patients, admitted in pediatric and adult/medical wards between January 2016 and February 2021. A majority of hospital deaths occurred in the second and third decades of life, following a hospital admission for a week. The overall mortality during 2016–2019 was 14% with little gender difference over time. Contrary to our expectations, the number of hospital deaths did not increase during the first year of the COVID-19 pandemic, between 2020 and 2021. The conclusion highlights the importance of longitudinal, socio-demo-graphic data on deaths as providing important insights for identifying ethical policy interventions focused on improving SCD outcomes over time, reducing inequities in access to care, and preventing what might be considered “excess” deaths.

## Introduction

With a decline in overall global infant and childhood mortality rates, cheaper and more accessible diagnostic tools, the incidence of single gene disorders, especially sickle cell disease (SCD), are projected to contribute significantly to pediatric morbidity, disability, and mortality in low- and middle-income countries (1). The pervasive health inequalities and the imminent scope for improving outcomes with known, simple interventions has been reiterated by the Lancet Hematology Commission (2). The sickle gene is one of the most common globin gene mutations in its homozygous (SS) and compound heterozygous forms, and 7% of the world’s population is estimated to be a carrier of its different mutations (3). The abnormal form of hemoglobin S results in hemolytic anemia and vaso-occlusion of small vessels causing varied clinical presentation and multi organ disease, resulting in 11 times higher mortality burden as compared to other diseases globally (1). Nigeria, India, and the Democratic Republic of Congo constitute half of the cases globally (4). India accounts for 14.5% of the global SCD newborns, estimated to be over 42,000 a year, second to sub-Saharan Africa (4–8). The highest prevalence is reported from Madhya Pradesh followed closely by Maharashtra, Gujarat, Odisha, and Kerala, respectively (9, 10).

Epidemiological modeling based on small, dated regional studies largely from sub-Saharan Africa estimate that between 50 and 90 percent of children affected by SCD will/die before the age of 5 years (5, 7, 11–13). These projections mask significant health disparities underpinning treatment outcomes and mortality across and within low and middle income countries, ignoring the dynamic shifts in healthcare systems over time (14). One underlying assumption in this literature is that treatment either is not accessible or not economically feasible. This premise, coupled with global health targets of reducing under 5 mortality (SDG 4), privileges public health interventions for screening and prevention of new births, at the cost of undermining investments in long-term/comprehensive and social care (15). This paper raises a specific question about the significance of the socio-demographic profile of patients dying of SCD in a hospital setting in India. In doing so, our objective is to identify factors associated with “excess” or potentially preventable mortality for SCD patients and, thereby, suggest ethical shifts in policy required to improve long term outcomes for children and adults, and their families struggling to access basic care.

It is important to note both homozygous (SS) and compound forms of SCD seem to be variable in their clinical manifestations across different parts of India. The extent to which such clinical variability reflects the specific genotype and/or the high fetal hemoglobin levels in the relevant populations continues to be debated. The role of social factors, poverty, malnutrition, and gender in particular, needs to be foregrounded in any clinical profiling of the disease. A complex picture of genetic variance and heterogeneity is accompanied by striking levels of poverty, inequalities in income and access to healthcare, especially in remote, rural parts of India still inhabited by 70% of the population. The impact of SCD on the lives of the poorest, socio-economically marginalized groups, designated as scheduled tribes (ST), scheduled castes (SC), and other backward classes (OBCs) has been highlighted by others (16). They constitute 25 percent of the population and live predominantly in remote, rural areas with limited access to healthcare facilities.

Despite the availability of cheap diagnostic tests and treatment options, including curative, stem cell transplants available across public and private sectors, SCD remains a largely neglected disease within healthcare policy and practice across India. The prevalence of sickle cell carriers among different *adivasi* (tribal) and scheduled groups varies from 1 to 40% (17). Rather than the prevalence rates *per se,* our concern here is with the wider social determinants of health and poor access to public healthcare facilities that exacerbate the adverse health outcomes for children born with SCD in these communities. The overlap between caste/ tribe/ religious minorities and inverse health outcomes is quite stark, as reiterated in the latest Family Health Survey figures for Maharashtra (18). Analyses of 2021 Census figures reveal that infant mortality (110), crude death rate (13) as well as low birth weight (40 percent) figures were nearly twice as high among the tribal communities as the population of India as a whole (19). What is especially important is that since 70 percent of healthcare is accessed within the private sector, out of pocket expenses for long-term conditions such as SCD is a major cause of family debt and poverty.

We know that new-born screening and the use of prophylactic penicillin and hydroxyurea, for example, reduce morbidity and mortality across high and low and middle-income settings (2, 20). In fact, a review from Tanzania suggests that such low-cost measures, alongside better education for parents and families, can reduce up to 70 percent of childhood mortality related to SCD (16). Despite a tacit consensus on high under 5 mortality caused by SCD in LMICs, there is little empirical data published on the clinical or socio-demographic profile of mortality related to SCD in India and other LMICs. A discussion on mortality might appear as part of clinical profile, treatment outcomes, hospital morbidity surveys (21, 22), case reports (23), perinatal outcomes (24, 25), or newborn cohort follow-up studies (25, 26). Insights into the importance of a social profiling of hospital deaths is exceptional (27–29). Following Serjeant et al. (28), we argue that longitudinal, socio-demo-graphic data on deaths related to SCD is essential for identifying interventions aimed at improving outcomes over time, reducing inequities in access to care, and preventing what might be considered “excess” deaths. This paper aims at bridging this gap in literature, acknowledging the long and arduous struggle of patients and parents for improving long term outcomes of care.

## Methods

This paper uses anonymized hospital records of 447 patients with SCD who died following admission for treatment in pediatric and adult/medical wards between January 2016 and February 2021, at the Government Medical College and Hospital (GMCH) Nagpur. Ethical permission was sought from the GMCH Ethics Committee. We used 2016 as a pragmatic temporal cut off point to ensure a degree of confidence in the consistency of coding in the records over time. Deaths in 2020–2021 were analyzed separately to draw out any changes related to COVID-19. In addition to the clinical features recorded at admission and cause of death, detailed data were obtained on demographic features such as gender, age, religion/caste, income and approximate distance between the home address and hospital. Income and distance from the hospital served as a proxy for socio-economic position, in conjunction with caste/tribe affiliation as noted in the hospital admission records.

Two important questions were asked in relation to the dataset: (i) what does the social profile or distribution (age, gender, and socio-economic background) of hospital deaths tell us about who dies of SCD in a hospital setting?, and (ii) can we draw any lessons from a longitudinal social profile of these deaths for improving treatment outcomes and reducing, what might be considered, avoidable mortality? Given our focus and small numbers in each category, a statistical analysis was not appropriate. Records of patients confirmed to have either homozygous SS or sickle compound heterozygous patterns (such as sickle-beta thalassaemia, on hemoglobin analysis by cellulose acetate electrophoresis and high-performance liquid chromatography), hospitalized for any morbid event during the period, either in pediatric or adult/medicine wards, were collated for analysis. The clinical information and cause of death were not included in analysis, given our focus on socio-demographic profiling of deaths. Further, in the absence of a research protocol, it was not possible to co-relate the clinical characteristics to socio-demographic features in an anonymized dataset.

## Results

Between January 2016 and January 2021, 3,778 patients received in-patient care in pediatric and adult medical wards at GMCH, Nagpur, diagnosed with sickle cell or sickle beta thalassemia (SBT, Table 1). Out of these, 682 were admitted during periods of strict lock-down policies imposed by Maharashtra government in response to COVID-19 pandemic. Mortality between 2016 and 2019: was 13.6% (*n*-423) which dropped to 3.5% during 2020–21 (n-24, Table 1). The overall mortality between 2016 and 21 was 11.8%. The admission records of these 447 patients who died were classified and analyzed by socio-demographic features, roughly estimated distance between home address and the hospital, length of in-patient stay (number of days between admission and death). Records of 33 heterozygous (AS) children and adults were excluded from analysis.

**Table 1:** Gender distribution of (N-3778) sickle cell admissions and (N-447) deaths (between Jan 2016 and Jan 2021).

Jan 2016 to Dec 2019					Jan 2020 to Jan 2021			
Admissions	3096	% (total deaths)	Male	Female	682	% (total deaths)	Male	Female
Mortality	*n* (%)				*n* (%)			
SS	401 (13%)	88.7 %	221	180	23 (3.4%)	82%	10	13
SBT	22 (0.7%)	4.9 %	9	13	1 (0.1%)	3.5%	1	0
Total	423 (13%)		230 (54.4%)	193 (45.6%)	24 (3.5%)		11 (45.8%)	13 (54.2%)

As seen in Table 2, between 2016 and 19, under 5 mortality was 19.1% (423 deaths). The highest incidence of death was in the age group of 6–10 years (32.2%), followed by 19–30 years (21.7%) and 11–18 years (20.3%), respectively. During 2020–2021, 24 deaths were recorded with none in children under 5 year. The majority (13) were in the age group 19–30 years (54.2%), followed by 11–18 (16.7%). Taking into account, the overall higher risk of infant and child mortality in the SC and ST populations, we expected more deaths in children under 5 years. Even though mortality was marginally higher in females (54.2%) during 2020–2021, it was conversely higher among males between 2016 and 19 (54.4%).

**Table 2:** Gender and age profile of patients who died between Jan 2016 and Jan 2021 (*the oldest patient in the dataset was 62 years).

		Jan-2016 to Dec-2019			Jan-2020 to Jan-2021		
Age/gender		*N* (%)	Male (%)	Female (%)	*N* (%)	Male (%)	Female (%)
Age (years)	0–5	81 (19.1)	39 (9.2)	42 (9.9)	0 (0)	0	0
	6–10	136 (32.2)	76 (18)	60 (14.2)	1 (4.2)	1 (4.2)	0
	11–18	86 (20.3)	49 (11.6)	37 (8.7)	4 (16.7)	1 (4.2)	3 (12.5)
	19–30	92 (21.7)	50 (11.8)	42 (9.9)	13 (54.2)	5 (20.8)	8 (33.3)
	31–39	15 (3.5)	10 (2.4)	5 (1.2)	3 (12.5)	1 (4.2)	2 (8.3)
	40+	13 (3.1)	6 (1.4)	7 (1.7)	3 (12.5)	3 (12.5)	0
	Total	423	230 (54.4)	193 (45.6)	24	11 (45.8)	13 (54.2)

Table 3 summarizes the religious/caste/tribe profile of patients who died during the study period. During the first year of COVID-19, 2020–2021, 50% of the patients who died were from the SC category, while ST, other backward classes (OBC) and *Vimukta jatis*, nomadic tribes (VJNT) contributed 16.7, 29.2, and 4.2%, respectively. This picture roughly corresponds to the overall patient population of the GMCH rather than the sickle cell population of the state as a whole.

**Table 3:** Socio-economic features of patients who died between Jan 2016 and Feb 2021 (*lower, middle, and upper socio-economic brackets use census categories for income of male head of the household).

		Jan-2016 to Dec-2019			Jan-2020 to Jan-2021		
Gender		*N* (%)	Male (%)	Female (%)	*N* (%)	Male (%)	Female (%)
Socio-economic status	LL	95 (22.5)	54 (12.8)	41 (9.7)	13 (54.2)	8 (33.3)	5 (20.8)
	LM	261 (61.7)	131 (31)	130 (30.7)	9 (37.5)	2 (8.3)	7 (29.2)
	M	4 (0.9)	4 (0.9)	0	1 (4.2)	1 (4.2)	0
	UM	17 (4)	12 (2.8)	5 (1.2)	1 (4.2)	0	1 (4.2)
	Unknown	46 (10.9)	29 (6.9)	17 (4)	-	-	-
Distance from GMC (km)	<20	242 (57.2)	123 (29.1)	119 (28.1)	9 (37.5)	5 (20.8)	4 (16.7)
	20–50	86 (20.3)	51 (12.1)	35 (8.3)	1 (4.2)	0	1 (4.2)
	50–100	35 (8.3)	19 (4.5)	16 (3.8)	3 (12.5)	2 (8.3)	1 (4.2)
	>100	59 (13.9)	36 (8.5)	23 (5.4)	10 (41.7)	4 (16.7)	6 (25)
	Unknown	1 (0.2)	1 (0.2)	0	1 (4.2)	0	1 (4.2)
Total		423	230	193	24	11	13

Table 4 presents a bird’s eye-view of the socio-economic background of the patient population of the GMCH, where nearly 83% of the admissions represent the lowest two income brackets. However, 77.5% of the patients who make it to the hospital and die there live within a radius of 20–50 km. As expected, a significant inverse relation can be seen between socio-economic status and mortality. During 2016–2019, patients from lower and lower middle classes represented 84.2%, rising to 91.7% (22 of the 24) in the last year of our analysis (2020–21). Even though our numbers are small, roughly calculated distance between the residential address and the hospital, and lack of access to private or public transport in rural areas are two factors determining access to secondary/tertiary care and treatment outcomes. Hence, between 2016 and 2019, a majority (57.2%) of the patients who died in our study lived within a radius of 20 km, 20.3% lived between 20 and 50 km away and only 14% lived up to a 100 km away from the GMCH. In comparison and contrary to our expectations, during the peak of COVID-19 (2020–2021), 41.7% patients who died in our sample lived about 100 km away and only one of these was attributed to COVID-19.

**Table 4:** Distribution by religion of patients who died between Jan 2016 and Dec 2019.

		*N* (%)	Male (%)	Female (%)
Religion	Hindu	297 (70.2)	158 (37.4)	139 (32.8)
	Muslim	7 (1.7)	4 (0.9)	3 (0.8)
	Buddhist	119 (28.1)	68 (16)	51 (12.1)
	Total	423	230	193

As suggested earlier, religion, caste/tribe affiliation remains another proxy indicator for socio-economic deprivation and health inequalities in India. For Maharashtra state, the SC households represent 17%, with ST and OBCs constituting 11 and 28% of the population, respectively (3). Unfortunately, we had to rely on the 2020–2021 break-up for caste/tribe affiliation, since, 2016–2019 records only included the religious break-up (Tables 4, 5).

**Table 5:** Caste distribution of patients who died between Jan 2020 and Jan 2021.

		*n* (%)	Male (%)	Female (%)
Caste categories	SC	12 (50)	5 (20.8)	7 (29.2)
	ST	4 (16.7)	3 (12.5)	1 (4.2)
	OBC	7 (29.2)	3 (12.5)	4 (16.7)
	VJ-NT	1 (4.2)	0	1 (4.2)
	Total	24	11	13

Table 6 shows that, during 2016–2019, a majority of patients (137) were admitted for 4–7 days and were between 6 and 10 years of age (missing data were reported for 166 patients). The longest stay of 84 days was for a patient aged 19–30 years; while 12 patients between the ages of 1 and 30 died within 24 h of admission (This data were not available for 2020–2021). This finding suggests that despite greater economic hardship and lack of easy access to public transport, patients do not simply come to the hospital at a terminal stage of emergency. Given our focus, we have not included the clinical features of the patients since we did not have a protocol enabling an appropriate analysis of the complications leading to death. It is worth noting, however, that the most frequent cause of death in the dataset (2016 and 2019) was severe anemia or hemolytic crisis (in nearly 24% cases) followed by sepsis, VOC/ACS; VOC/stroke, hepatic encephalopathy/failure, and renal failure. We found surprisingly low hemoglobin levels for some of the younger patients– the lowest being 2.7 gm/dL (age group <10 years) and 3.7 (> 40 years). The levels of nutritional anemia and co-morbidities being higher in the lower socio-economic groups might be a confounding factor that needs further research.

**Table 6:** Duration of hospital stay by age between 2016 and 2019 (^*^calculated as the interval between the date of admission and death; data not available for 2020–2021).

No. of days in hospital before death									
Age group	No records	1–3 days		4–7 days	8–14 days	15–30 days	31 or more	Min days	Max days
		24 h							
0–5	31	(3)	10	23	13	4	0	1	24
6–10	47	(4)	12	53	16	8	0	1	22
11–18	36	(2)	12	29	7	2	0	1	19
19–30	42	(3)	13	22	7	7	1	1	84
31–39	6	-	0	6	3	0	0	0	0
40 and above	4	-	3	4	2	0	0	2	14
Total (423)	166		50	137	48	21	1		

## Discussion

The socio-demographic profile of patients in this study broadly reflects the SCD patient population of central India (19). It is important to mention that Maharashtra generally performs better on gender and health indices than other central and North Indian states. This reinforces the importance of regional context in analysis of SCD mortality as well the significance of longitudinal studies over time. For instance, extreme weather conditions and rainy season are likely to cause higher morbidity and mortality among children in India, implying regional variations (30, 31, 32). Despite robust evidence about gendered inequalities in nutrition, access to healthcare and related higher infant/female mortality (10), a gender bias is not consistent in our and other hospital-based studies. There are a couple of papers suggesting a male bias in accessing hospital care for SCD patients (10, 33). During 2016–2019, male mortality was slightly higher (54.4%) in our dataset when the overall male admissions were also higher. Conversely, both admissions and mortality figures were marginally higher in females (54.2%) during 2020–2021. We need more longitudinal studies of gender and age distribution at diagnosis, as well as clinical features at admission for a nuanced analysis of the impact of caring and cultural practices on SCD related deaths related to SCD.

Since literature suggests that nearly half of SCD patients die at home (27, 33), our data are likely to present a partial picture of the numbers and social profile of deaths in this population. Equally, Table 2 can be misleading in suggesting a rising trend in mortality over the years. Rather, it represents poor record keeping in an under-resourced public hospital or a change in data codes, posing challenges for secondary analysis. Likewise, the fewer deaths reported during the COVID year reflect the practical, financial, and social constraints in accessing the hospital by patients who lived further away and relied on public transport. Access to state healthcare, across the board, was severely constrained and admissions to designated COVID hospitals were risky both clinically in terms of potential contagion in the community and stigma for the family. Some of the *gram panchayats* (village councils) operated strict local quarantine policies in parts of Maharashtra, not allowing people back into the village if they had been in a hospital. Following a hospital stay. Sadly, in one such case, a child died in his father’s lap, following discharge from GMCH in 2020 (personal communication, DJ with the parent).

As reflected in Table 6, data on the length of hospital stay before death points to the fact that both pediatric and adult patients from rural and urban areas are seeking tertiary care before they are in a critical state. Two of the older surveys conducted by the Indian Council of Medical Research (ICMR) from Gujarat (Western India) reported that 20 per cent of children with sickle disease died by the age of two, while 30 percent children with SCD from the *adivasi* (ST) groups did not reach adulthood (34, 35). Going back to our main research question about the incidence of under 5 mortality, our study suggests that a majority of SCD related deaths in the hospital occur in the second (16.7%) and third (54.2%) decades of life. In the absence of universal newborn screening, we do not know whether the low under 5 mortality in our data masks unreported deaths in the community of children who remain undiagnosed. Other studies, including Purohit et al. (36), however, corroborate our findings. Shah et al. (37), for instance, based on hospital autopsy reports of 25 SCD patients, concluded that the mean age at death was 30 years and peak mortality was in the second to fourth decades of life.

Sheshadari et al. (27) are exceptional in their socio-demographic analysis of a cohort of 157 patients, enrolled in their comprehensive community care program in a remote, *adivasi* area of Gudalur (South India). Their cohort included children screened at birth and followed up for 10 years. The median age of death due to SCD was 24.5 years in contrast with 55 years for the population at large. Hence, even though SCD was a significant factor for premature death by 25 years, there were no deaths under 5 years in their cohort. A declining trend of mortality was noted between 2008 and 2018, highlighting the positive impact of newborn screening and community-based interventions in care. This is the only study we found using verbal autopsy methods to profile SCD related deaths within the community. This method is important since death certificates often do not mention the cause of death if a person dies in a non-hospital setting in India.

Our data from 2020 to 21 (Table 3) show that 50% of the 24 patients who died belonged to SC category, while ST, OBC, and VJNT category contributed to 16.7, 29.2, and 4.2%, respectively. While these figures are small and seem to support the contentious hypothesis of higher prevalence of SCD in SC, ST and similar disadvantaged groups, socio-economic status is an important confounding factor that self -selects the patient population at a busy state hospital. A significant association can be seen between socio-economic status and mortality. Out of 24, 13 patients (54.2%) belonged to the lower class and 9 (37.5%) belonged to the lower-middle class. Similarly, during 2016–2019, 22.5% of the 423 deaths were from lower-lower and 61.7% from the lower-middle class groups. Anecdotal evidence suggests that upper class/caste SCD patients are more likely to access private healthcare facilities and are, therefore, often excluded from the community-based surveys targeting particular lower socio-economic groups. In our study, more than 40% of the patients who died resided more than 100 km away from the tertiary care hospital (Table 3). Distance from the hospital and access to transport have been reported as adversely affecting treatment outcomes and mortality among SCD patients in Brazil (31, 38) even though we did not have details of the socio-economic position of the patients’ household in our study, it is closely related to structural inequities in health and SCD outcomes as reflected in the longitudinal study by Sheshadari et al. (27).

One of the surprising findings in our dataset is the number of deaths in AS patients, 29 out of 452 (6.4%) during 2016–2019 and 4 out of 20 (14.5%) in 2020–2021. There is surprisingly little discussion on the significance of the clinical differentiation between SS and AS in relation to deaths in the literature in India. It is important mention that, given the sheer number of sickle cell carriers across India, and the wider policy campaigns for a “sickle free India,” carriers as a clinical category as well as the subject of prevention policies is an under researched field in India.

We acknowledge the limitations of our study. Firstly, due to incomplete digitization of patient records in the past, we had to manually extract data for some periods from anonymized hospital admissions to the adult and pediatric wards. The codes and data categories often did not match over time, resulting in significant number of missing data, including details of vaccination and hydroxyurea records. In the absence of a research protocol, it was not possible to co-relate the baseline clinical features or the cause of death to the outcomes. Similarly, due to gaps in records on clinical data, any comparison between patients who died and those who survived would not be valid. Despite these limitations, we can safely conclude that a majority of SCD patients died in their second and third decades of life, having been in hospital, on an average, for a week, irrespective of their age.

## Concluding observations

There are very few studies outlining the social-demographic profile of patients who die of SCD in India. Given the importance of the healthcare system and policy context (16, 29), broad cross-national comparisons of cohorts are unhelpful. We know that morbidity and mortality patterns are likely to vary across low and middle-income countries, depending on the policy interventions for SCD as well as the scale of structural inequalities in access to health. Due to lack of access to transport and poor literacy, nearly half the number of patients who die of SCD do not make it to a hospital. Our findings and recent literature do not support the policy assumption that a majority of SCD patients die before the age of 5 years or that it is predominantly a “tribal disease.” As suggested in the introduction, high under 5 mortality is often used to justify public health interventions that prioritize carrier screening with a view to preventing new births as a cheaper option, rather than investing in improving outcomes and reducing mortality. Recently, Government of India pledged to eradicate sickle cell from India by 2047 (https://www.financialexpress.com/budget/budget-2023, Accessed October 22, 2023). The practical limitations and the complex ethical reverberations, of preventing new births of children with a genetic disorder, for reproductive choice and disability rights, are conveniently sequestrated (39).

As an increasing number of children with SCD are surviving into adulthood, we argue for a shift in policy focus to improving long -term clinical care and supporting their educational and employment needs. New-born screening alongside an intensive follow-up care plan have shown promising results both at GMCH Hospital, Nagpur (30, 40) and in some of the remote, rural *adivasi* areas such as Gudalur (27) and South Gujarat (25) as elsewhere in low and middle income countries across the world (41). Digital technology, remote clinical networks and better professional and patient education can all contribute to improving outcomes of care, as highlighted recently by the Lancet Hematology Commission on SCD (2). A good example is the development of a basic information toolkit that we produced in consultation with regional teams of clinicians, patients and family carers, at a minimal cost. It is now available, free to download, from the ASHWINI hospital website, the host training institution, with a version for patients and family carers translated into seven languages (http://scd.ashwini.org/pro/; http://scd.ashwini.org/family/). We need to think more creatively in devising local, context-specific solutions for improving knowledge and practice that can have a ripple effect on improving long-term outcomes for adults while reducing SCD morbidity and mortality in children, rather than waiting for the unfolding of policy rhetoric on zero births of children with sickle cell by 2047.

## Data availability statement

The original contributions presented in the study are included in the article/supplementary material, further inquiries can be directed to the corresponding author.

## Ethics statement

The studies involving humans were approved by Chair, Ethics Committee, Government Medical College and Hospital Nagpur, India. The studies were conducted in accordance with the local legislation and institutional requirements. Written informed consent for participation was not required from the participants or the participants’ legal guardians/next of KK in accordance with the national legislation and institutional requirements.

## Author contributions

SC: Conceptualization, Data curation, Formal Analysis, Methodology, Supervision, Validation, Writing – original draft, Funding acquisition, Project administration, Writing – review & editing. DJ: Conceptualization, Data curation, Formal Analysis, Investigation, Methodology, Supervision, Validation, Writing – review & editing, Resources, Writing – original draft. NN: Data curation, Formal Analysis, Investigation, Methodology, Writing – original draft, Resources. RS: Data curation, Formal Analysis, Investigation, Resources, Writing – original draft.
